# Primary hepatic neuroendocrine tumor associated with hypertension: A case report

**DOI:** 10.3389/fsurg.2022.1021806

**Published:** 2023-02-17

**Authors:** Bin Zhao, Jie Mao, Yumin Li

**Affiliations:** Department of General Surgery, Lanzhou University Second Hospital, Lanzhou, China

**Keywords:** case report, hypertension, primary, liver, neuroendocrine tumors

## Abstract

**Background:**

Primary neuroendocrine tumors are exceedingly rare and often misdiagnosed. The combined methods of ultrasonography, computed tomography ,and magnetic resonance imaging are typically applied. The diagnosis of the disease mainly depends on the histopathological examination. Surgical resection is the most effective treatment.

**Case presentation:**

In the report, we describe the case of a patient with a primary hepatic neuroendocrine tumor (PHNET) associated with hypertension. The patient suffered from uncontrolled hypertension before the operation, and the blood pressure was not well controlled by oral antihypertensive drugs such as nifedipine, valsartan, and hydrochlorothiazide, but the patient's blood pressure completely returned to normal after the operation without drug control.

**Conclusions:**

We encountered a rare case of a PHNET associated with hypertension *via* careful screening noticed by the patient at work; furthermore, we hope to collect more cases and find the relationship between neuroendocrine tumors and hypertension.

## Background

Primary neuroendocrine tumors are those derived from embryonal neural crest cells, also called Argentaffin or Kulchitsky cells, which have the potential function of regulating hormone secretion (1, 2). These tumors can arise from many locations, including in the gastrointestinal (GI) system, accounting for 55%; the bronchus or lung, accounting for 30%; and other organs such as the pancreas (2%), biliary system (1%), and reproductive system (1%) (3). Primary hepatic neuroendocrine tumors (PHNETs) are very rare, and the first case was reported in 1958 (4). Early detection of an NET is unachievable unless metastases are observed or the tumor is resected, and it can be misdiagnosed as hepatic malignancies such as hepatocellular carcinoma or cholangiocarcinoma or as hepatic metastases due to a lack of histological confirmation. The sites of metastases predominantly include the lymph nodes and liver. Hepatic neuroendocrine tumors are divided into primary and secondary tumors, and the latter are usually metastatic from gastric neoplasms.

## Case presentation

Here, we report the case of a patient with a PHNET that was incidentally discovered due to severe hypertension. A PHNET was not considered initially since no clinical symptoms were observed but was diagnosed after the histopathological analysis.

**Figure 1:**
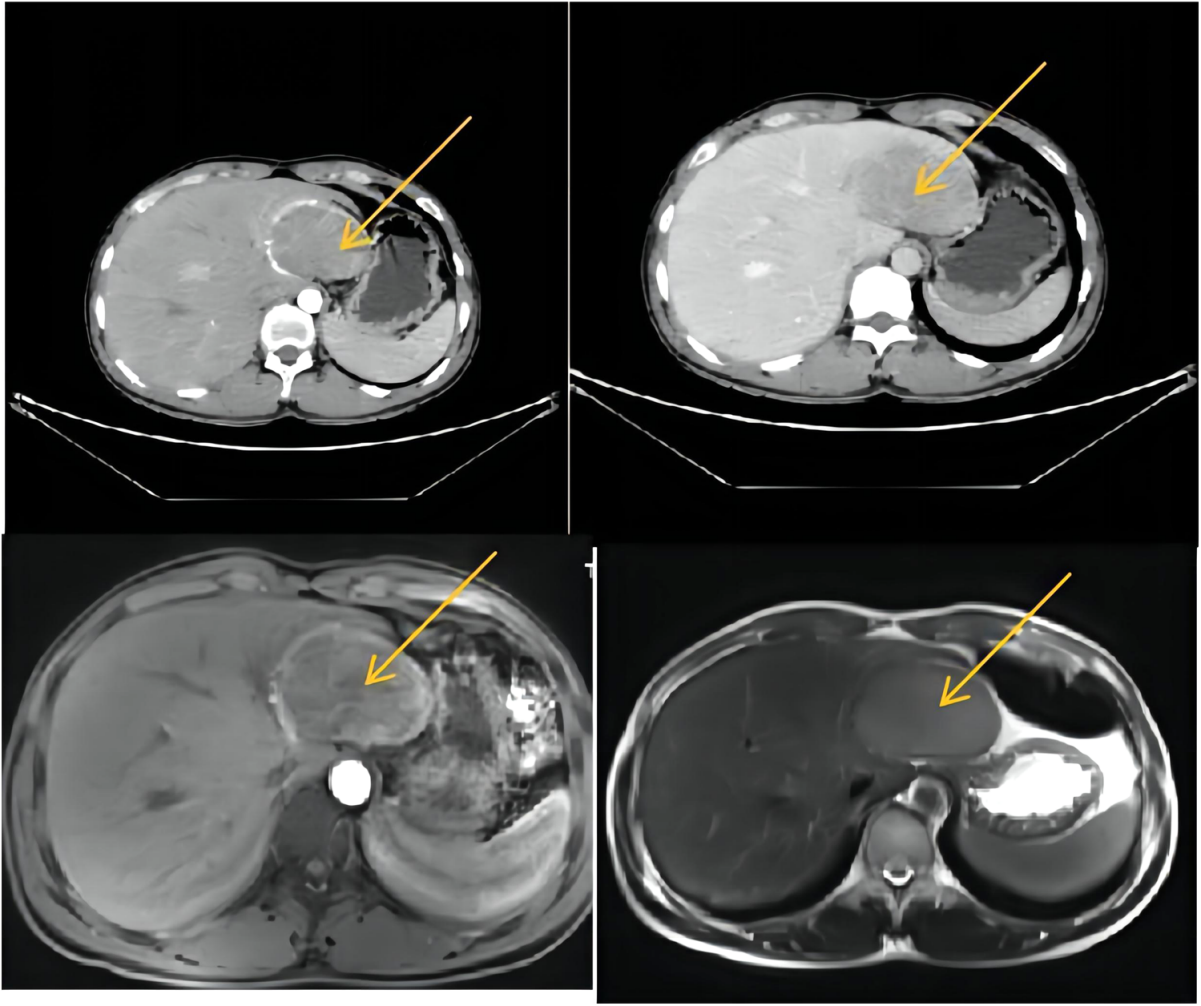
CT and MRI (a round solitary hepatic mass in the left lobe of liver was found, measuring 7.5 cm × 6.1 cm × 6.2 cm, with clear and smooth border and homogeneous density in enhanced scan). CT, computed tomography; MRI, magnetic resonance imaging.

A 26-year-old male was admitted to Lanzhou University Second Hospital with a 2-year history of hypertension. His blood pressure (BP) went up to 280/170 mmHg, and he needed to take a quadruple dose of antihypertensive drugs to control hypertension. There was no history of hepatitis, Cushing syndrome, diabetes, heart diseases, carcinoids, or other remarkable diseases. In terms of the physical examination, the patient was anicteric with a soft abdomen. The laboratory test results were all normal. The tumor markers α-fetoprotein (AFP), carcinoembryonic antigen (CEA), CA19-9, and CA125 were all within normal ranges. The thyroid hormone level is normal. Vanillylmandelic acid (VMA), urine17-ketosteroide (17-ks), and 17-hydroxy-cortico-steroid (17-OHCS) were within normal ranges. The serum cortisol level was normal. With the use of a liver-specific contrast agent, an abdominal contrast-enhanced computed tomography (CT) was performed, and a round, solitary hepatic mass in the left lobe of the liver was observed that measured 7.5 cm × 6.1 cm × 6.2 cm and had a clear and smooth border as well as homogeneous density as seen on the enhanced scan. A proper hepatic artery branch had enveloped the lesion in the arterial phase. There was no evidence of other diseases or lymphatic metastasis. Enhanced magnetic resonance imaging (MRI) showed an oval mass with a high signal intensity that measured 7.7 cm × 5.1 cm × 6.6 cm (Figure 1). An atypical hepatic adenoma was considered. Hypointense and homogenous lesions were shown on preenhanced T1- and T2-weighted images. The Apparent Diffusion Coefficient (ADC) values observed with diffusion-weighted imaging (DWI) were restricted. The lesions appeared to have capsule enhancement in the portal phase and delayed phase, and obvious enhancement washout was observed on the delayed images. Abdominal ultrasound imaging demonstrated a mass in the left lateral segment of the liver, which was defined as hepatic hemangioma. No other occupying lesions were found on systemic CT scan. A diagnosis of hepatic carcinoma (tumor) was made, and further immunohistochemical testing was needed to confirm the type of tumor.

## Gross findings

**Figure 2:**
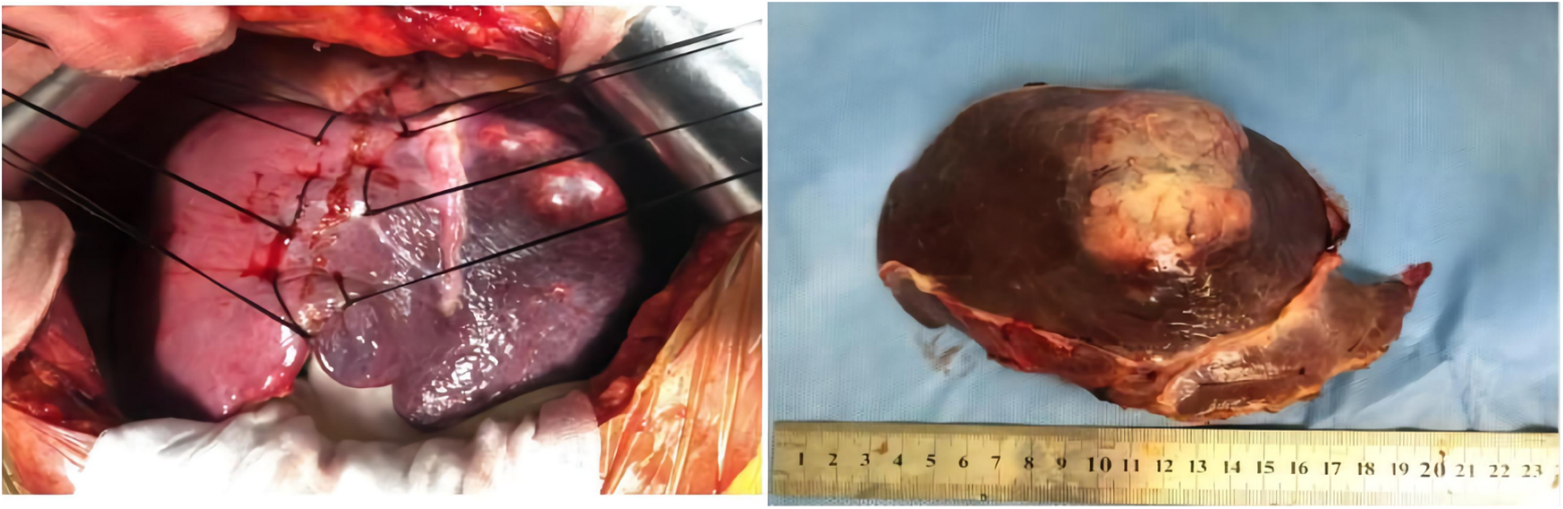
Left hepatectomy was conducted, and a single exophytic nodule was observed in the left lobe of the liver. No metastases were identified upon exploration. Grossly, the section of tumor appeared as a grey-white and dusty-red well circumscribed solid, which was 7.5 cm × 7 cm × 5 cm in size, and it has intact capsule and a clear border with hepatic tissue.

After multiple discipline discussions and the blood pressure was well controlled, left hepatectomy was conducted, and a single exophytic nodule approximately was observed in the left lobe of liver. No metastases were identified upon exploration. Grossly, the tumor section appeared as a gray-yellow and dusty-red solid mass that was well circumscribed and was 7.5 cm × 7 cm × 5 cm in size, and it had an intact capsule and a clear border with hepatic tissue (Figure 2).

## Microscopic findings

The tumor cells with consistent sizes were arranged as solid nests and were funicular, gland-like structures; the blood sinus was rich in the nests among neoplastic cells. The cells had an abundant cytoplasm with a pinkish color, and the borders of the tumor cells were clear. The tumor cells had round or ovoid nuclei that were slightly enlarged, a dark cell nucleus, light cell cytoplasm, and a low mitotic rate. A clear demarcation between the tumor and surrounding liver tissue was observed.

**Figure 3:**
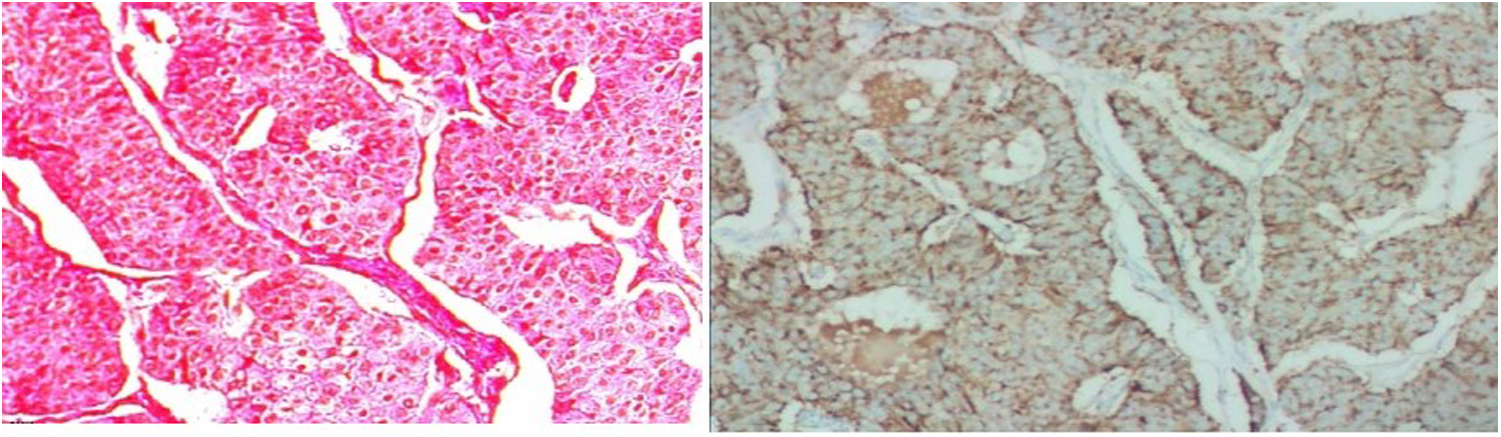
Histology of PHNET. The tumor cells with consistent size arranged as solid nests, funicular, gland-like structure, and blood sinus was rich in the nests among neoplastic cells; their abundant cytoplasmic with pinky color, and the borders of the tumor cells were clear. Tumor cells were round or ovoid nuclei and slightly enlarged, dark cell nucleus and light cell cytoplasm, lower mitotic rate. A clear demarcation between the tumor and surrounding liver tissue was observed. PHNET, primary hepatic neuroendocrine tumor.

The pathological diagnosis of neuroendocrine tumors was made, and the tumors measured 7.5 cm × 7 cm × 5 cm in size. The mitosis rate was 2 mitoses per 10 high-power fields. Hematoxylin-eosin (HE) staining was performed with the peroxidase-conjugated dextran polymer complex: liver tumor cells revealed Syn (+), CgA (+), CD56 (+), CK8/18 (+), CK (+), hepatocyte (−), and AFP (−). Vessels of the tumors showed CD34 (+) and positivity for Ki-67 > 3%. Syn, CgA, CD56, CK8/18, and CK were positive in the liver tumor cells, while other markers, including hepatocytes and AFP, were negative. CD34 was positive in the vessels of the tumor, and positivity was found for Ki-67 more than 3%. It was classified as G2 (Figure 3).

## Conclusions

**Figure 4:**
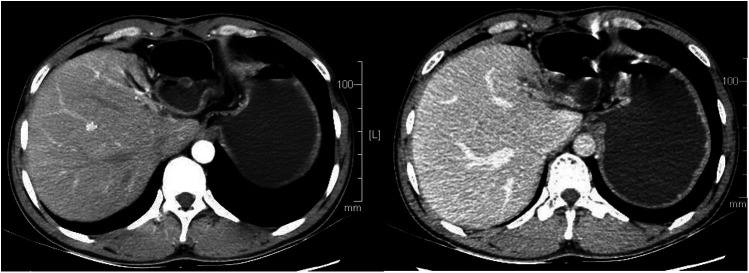
The patient had a CT scan 1 year after the operation, and no recurrence was found. CT, computed tomography.

The patient recovered well after operation, and the blood pressure returned to normal without drug control. He was cured and discharged 1 week after operation. One year after operation, the patient has no abdominal pain and abdominal distension, There was no recurrence on enhanced CT during reexamination, as shown in Figure 4.

## Discussion

PHNETs are exceedingly rare, for its absence of specificity and low incidence rate. The disease can be easily misdiagnosed due to the fact that its clinical features are common to hepatic hemangioma, hepatocellular carcinoma, or cholangiocarcinoma. Therefore, the diagnosis of PHNETs is still a debatable point, and the final diagnosis depends on careful analysis and pathological examination by surgery. No PHNET associated with hypertension in the literature has been reported. In our case, accelerated hypertension was diagnosed and PHNET was incidentally found the highest blood pressure even 280/170 mmHg, yet sometimes fail to get BP under control despite having antihypertensive medications intensified. After surgery, the blood pressure went down to a normal level without taking any antihypertensive drugs. It showed that PHNETs may aggravate hypertension. It is speculated that the occurrence and severity of hypertension may correlate with the elevation of serotonin or the metabolites 5-hydroxyindoleacetic acid released by NET (5, 6).

Some previous studies reported that primary hepatic neuroendocrine tumors mostly happened in middle-aged patients and more frequently happened in females. Right lobe incident rate is higher than that of the left lobe. In our study, the patient was 26 years old and had no manifestation of carcinoid syndrome other than hypertension.

An abdominal ultrasound contrast was performed and an echogenic lesion in the left hepatic lobe arterial phase as well as peripheral nodular arterial enhancement was detected, enhanced earlier than the liver parenchyma. The persistent enhancement was found and the lesions were enhanced centripetally in the portal phase that resembled hepatic hemangioma. From our experience, Doppler ultrasonography can be used as preliminary screening of PHNETs, to distinguish the blood flow in lesion tissue from normal tissue, same inference with Piscaglia (6). CT and MRI scans showed a tumor characterization and clearly demonstrated a round ill-defined density mass, with a low density area inside. PHNETs are enhanced on the portal venous phase and the delayed phase, and the major lesion was surrounded by few satellite nodules. CT and MRI scans reflect the histological features of PHNETs that need careful imaging, or may be misdiagnosed as hepatic hemangioma, same as the results of Akahori et al. (7).

The treatment of PHNET mainly include: (1) hepatectomy, (2) hepatic regional therapy (arterial embolization, radioembolization, and chemoembolization), (3) local ablative therapy (radiofrequency ablation (RFA), cryotherapy, and microwave), and (4) interferon therapy. Surgical resection was mostly effective and used by hepatectomy and lymphadenectomy (8). For patients with metastatic liver, interventional therapy by arterial embolization, chemoembolization, or radioembolization was adopted to reduce lesions; an alternative therapy could be local ablative by RFA, cryotherapy, and microwave, so as to strive for the opportunity of surgery, but its benefit is debatable (5, 9, 10). Also, liver transplantation in the treatment of PHNETs acquired a certain therapeutic effect from the study of Alekseev et al. (11). In our case, circumscribed tumor lesions without metastasis in the liver, resection of lesions is a preferable treatment.

By using pathology and the 2019 WHO classification of PHNET, our case was defined as G2, well-differentiated NET (12), which has a good prognosis. The tumor cells were arranged as solid nests, consistent size, funicular, Gland-like structure, and revealed the same character with that reported by Sun et al. (13).

Either primary or secondary hepatic carcinoids are effective when treated by surgical treatment, with the 5-year survival rate for PHNET being 74%–78% and a 5-year recurrence rate of 18% after hepatectomy (9, 14–16). Shin et al. (17) reported from postoperative follow-up that surgical treatment is effective either on primary hepatic neuroendocrine tumors or secondary hepatic neuroendocrine tumors. In our case, there is no clinical sign of recurrence or metastasis of the tumor 24 months after surgery.

The current literature reports that secondary hypertension caused by neuroendocrine tumors is related to the following factors: (1) pheochromocytoma, with a prevalence of 0.2%–0.6% (18); (2) ectopic adrenocorticotropic hormone (ACTH) tumors, most of which are complicated with Cushing's syndrome (19); (3) pituitary growth hormone tumor (somatotropinoma), such patients have acromegaly in adulthood (20); (4) parathyroid adenoma, most of these patients are complicated with hyperparathyroidism (21).

The patient was hospitalized because of high blood pressure, and further examination revealed liver tumor. After resection of liver tumor, the patient's blood pressure accidentally returned to normal without drug control. This is a very rare and interesting case. The patient's endocrine hormone level was normal, he had no endocrine related complications, the relationship between hepatic neuroendocrine tumor and hypertension is not clear yet, with the lack of enzymes or catabolite proofs, and more such cases will need to be collected for further research in the future.

## Data Availability

The original contributions presented in the study are included in the article/Supplementary Material, further inquiries can be directed to the corresponding author.
